# Tolerance to the antinociceptive effects of chronic morphine requires c-Jun N-terminal kinase

**DOI:** 10.1186/s12990-015-0031-4

**Published:** 2015-06-12

**Authors:** David J. Marcus, Michael Zee, Alex Hughes, Matthew B. Yuill, Andrea G. Hohmann, Ken Mackie, Josée Guindon, Daniel J. Morgan

**Affiliations:** Department of Anesthesiology, Penn State College of Medicine, 500 University Drive, Room C2850, Mailcode H187, 17033 Hershey, PA USA; Department of Pharmacology, Penn State College of Medicine, 17033 Hershey, PA USA; Department of Psychological and Brain Sciences, Indiana University, 47405 Bloomington, IN USA; Gill Center for Biomolecular Science, Indiana University, 47405 Bloomington, IN USA; Department of Pharmacology and Neuroscience, Texas Tech University Health Sciences Center, 3601 4th Street STOP 6592, 79430 Lubbock, TX USA

**Keywords:** Morphine, Fentanyl, JNK, GPCR, GRK, Cisplatin, Desensitization, Tolerance, Mu opioid receptor, Chemotherapy

## Abstract

**Background:**

Morphine and fentanyl are opioid analgesics in wide clinical use that act through the μ-opioid receptor (MOR). However, one limitation of their long-term effectiveness is the development of tolerance. Receptor desensitization has been proposed as a putative mechanism driving tolerance to G protein-coupled receptor (GPCR) agonists. Recent studies have found that tolerance to morphine is mediated by the c-Jun N-terminal Kinase (JNK) signaling pathway. The goal of the present study was to test the hypotheses that: 1) JNK inhibition will be antinociceptive on its own; 2) JNK inhibition will augment morphine antinociception and; 3) JNK mediates chronic tolerance for the antinociceptive effects of morphine using acute (hotplate and tail-flick), inflammatory (10 μl of formalin 2.5 %) and chemotherapy (cisplatin 5 mg/kg ip once weekly)-induced neuropathic pain assays.

**Results:**

We found that JNK inhibition by SP600125 (3 mg/kg) produces a greater antinociceptive effect than morphine (6 mg/kg) alone in the formalin test. Moreover, co-administration of morphine (6 mg/kg) with SP600125 (3 mg/kg) produced a sub-additive antinociceptive effect in the formalin test. We also show that pre-treatment with SP600125 (3 or 10 mg/kg), attenuates tolerance to the antinociceptive effects of morphine (10 mg/kg), but not fentanyl (0.3 mg/kg), in the tail-flick and hotplate tests. Pre-treatment with SP600125 also attenuates tolerance to the hypothermic effects of both morphine and fentanyl. We also examined the role of JNK in morphine tolerance in a cisplatin-induced model of neuropathic pain. Interestingly, treatment with SP600125 (3 mg/kg) alone attenuated mechanical and cold allodynia in a chemotherapy-induced pain model using cisplatin. Strikingly, SP600125 (3 mg/kg) pre-treatment prolonged the anti-allodynic effect of morphine by several days (5 and 7 days for mechanical and cold, respectively).

**Conclusions:**

These results demonstrate that JNK signaling plays a crucial role in mediating antinociception as well as chronic tolerance to the antinociceptive effects of morphine in acute, inflammatory, and neuropathic pain states. Thus, inhibition of JNK signaling pathway, via SP600125, represents an efficacious pharmacological approach to delay tolerance to the antinociceptive effects of chronic morphine in diverse pain models.

## Background

Morphine and fentanyl are two opioid drugs commonly used for the treatment of pain [1]. Both compounds elicit their primary analgesic effects through activation of the μ-opioid receptor (MOR), a member of the G protein-coupled receptor (GPCR) superfamily. MOR is one of the most studied members of this family due to its relevance in pain management and dependence [2, 3]. Despite the clinical utility of MOR agonists, unwanted side effects such as respiratory depression, dependence and the rapid development of pharmacological tolerance limit the long-term clinical use of these analgesics in the outpatient setting [4].

Several mechanisms have been proposed for the development of tolerance to the antinociceptive effects of morphine. The desensitization of MOR signaling through its uncoupling from cognate effector pathways has been suggested to be a primary mechanism driving tolerance to opioid analgesics. Multiple studies have demonstrated the loss of MOR-effector coupling following chronic treatment with agonist [4–7]. Desensitization of MOR involves agonist-specific phosphorylation at C-terminal threonine 370 and/or serine 375 [8]. Phosphorylation at these residues is mediated primarily by GRK 2 and/or GRK 3 and causes the recruitment of β-arrestin 2 [8, 9]. Interaction between the phosphorylated MOR and β-arrestin 2 uncouples MOR from its associated G proteins and is also required for clathrin-mediated endocytosis [4, 10, 11]. Internalized MOR can be trafficked to the lysosome for degradation or can undergo dephosphorylation, leading to resensitization and recycling to the plasma membrane [9, 12]. Several other possible mechanisms have been implicated in the development of tolerance to opioid analgesics including the presence of delta opioid receptors [13, 14], glutamate signaling through NMDA receptors [15], nitric oxide signaling [16], protein kinase C activity [17], as well as down-regulation of MOR [4, 18].

Recent studies have demonstrated that JNK is involved in regulation of MOR signaling and tolerance for some opioid agonists. SP600125 is an anthrapyrazolone compound that inhibits JNK1, JNK2, and JNK3 with similar high affinity (Ki = 0.19 μM) [19]. Further, SP600125 exhibits >20 fold selectivity for JNKs compared to other MAPK pathway family members and also >100 fold selectivity relative to more distantly related kinases such as protein kinases A and C. SP600125 exhibits efficacy in cells (where it dose dependently inhibits c-Jun phosphorylation) and in animals (where it can block lipopolysaccharide (bacterial) induced expression of tumor necrosis factor-alpha) [19]. Recently, pre-treatment with SP600125, has been shown to block acute tolerance to the antinociceptive effects of morphine, morphine-6-glucoronide, and buprenorphine in mice [20]. Conversely, acute tolerance to fentanyl-induced antinociception was not affected by inhibition of JNK signaling, but was absent in GRK3 knock-out (KO) mice [20]. These data demonstrate functional selectivity in opioid tolerance mechanisms differentially involving JNK and GRK signaling mechanisms [20]. Chronic SP600125 treatment prevents the development of chronic tolerance to antinociceptive [15, 21], as well as antiallodynic effects of morphine in a sciatic nerve injury model of neuropathic pain [22].

The present study utilized different pain models to evaluate the role of JNK signaling in the development of morphine tolerance in mice. First, we tested the hypothesis that SP600125 treatment alone elicits dose-dependent antinociception in the formalin test, an animal model of inflammatory pain. Second, we examined whether SP600125 treatment enhances morphine antinociception in the formalin test. Third, we determined the contribution of JNK signaling to the development of antinociceptive tolerance to both morphine and fentanyl in acute pain models (tail-flick and hotplate) and examined JNK’s role in tolerance to the hypothermic effects of morphine and fentanyl. Fourth, we determined whether JNK signaling enhances antinociception and mediates tolerance to the anti-allodynic effects of chronic morphine in a model of chemotherapy-induced neuropathy. Together, the results of this study show that JNK signaling causes analgesia and prevents tolerance to chronic morphine in both nociceptive and neuropathic pain models. Our findings demonstrate that targeting the JNK pathway represents a novel approach for enhancing antinociception and prevents behavioral tolerance for certain opioid analgesics such as morphine.

## Results

### Antinociceptive effect of SP600125 in the formalin test

**Fig. 1:**
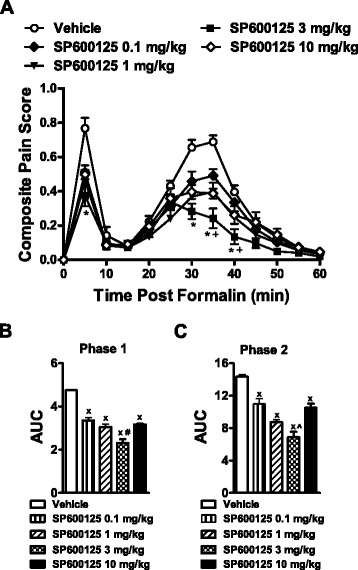
Antinociceptive effect of SP600125 in the formalin test. SP600125 suppresses formalin-induced pain behavior (**a**) in a dose-dependent manner. The four different doses of SP600125 (0.1, 1, 3 and 10 mg/kg) decrease the area under the curve (AUC) of (**b**) Phase 1 and (**c**) Phase 2 pain behavior. Data are expressed as mean ± S.E.M. (n = 5–6 per group). *p < 0.0001 for SP600125 different doses vs. vehicle group (ANOVA); + *p* < 0.017 vs. SP600125 (0.1 mg/kg) group (ANOVA, Bonferroni post hoc); x *p* < 0.0001 vs. vehicle group (ANOVA); #*p* < 0.039 for SP600125 (3 mg/kg) vs. SP600125 (0.1 mg/kg) (ANOVA, Bonferroni post hoc); ^*p* < 0.048 for SP600125 (3 mg/kg) vs. SP600125 (0.1, 1 or 10 mg/kg) (ANOVA, Bonferroni post hoc)

All four doses of SP600125 (F_4,21_ = 40.93, *p* < 0.0001), administered systemically (i.p.), suppressed composite pain scores relative to the vehicle-treated group in a time-dependent manner (F_44,231_ = 3.49, *p* < 0.0001) (Fig. 1a). This suppression was observed at 5 min (acute phase 1) and from 30 to 40 min (inflammatory phase 2) post-formalin injection (*p* < 0.0001). Composite pain scores were also lower for the SP600125 3 mg/kg dose relative to 0.1 mg/kg dose from 35 to 40 min post-formalin injection (*p* < 0.017), suggesting that 3 mg/kg SP600125 produces greater antinociception (Fig. 1a). Analysis of the Area Under the Curve (AUC) of pain behavior revealed that all doses of SP600125 produced antinociception relative to vehicle in both Phase 1 (F_4,21_ = 20.53, *p* < 0.0001) and Phase 2 (F_4,21_ = 42.23, *p* < 0.0001) of the formalin test (Fig. 1b and c). In the first phase, a higher dose of SP600125 (3 mg/kg) produced a greater antinociceptive effect relative to the lowest dose (0.1 mg/kg) (*p* < 0.039). In the second phase, SP600125 at 3 mg/kg produced a greater antinociceptive effect than all the other doses (*p* < 0.048).

### Antinociceptive effects of morphine, SP600125 and their combination in the formalin test

**Fig. 2:**
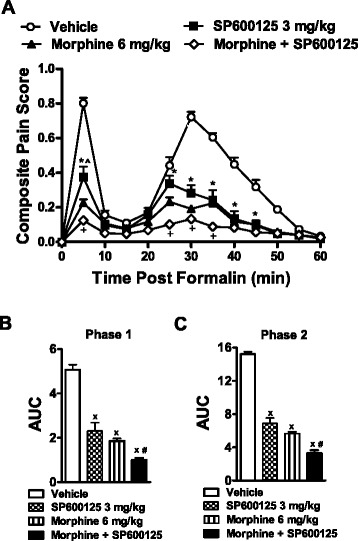
Antinociceptive effects of morphine, SP600125 and their combination in the formalin test. Sub-additive antinociceptive effects of the combination of SP600125 (3 mg/kg i.p.) with morphine (6 mg/kg i.p.) is observed for (**a**) the composite pain score and area under the curve (AUC) of (**b**) Phase 1 and (**c**) Phase 2 pain behavior. Data are expressed as mean ± S.E.M. (n = 5–6 per group). *p < 0.0001 for SP600125 different doses vs. vehicle group (ANOVA, Bonferroni post hoc); + *p* < 0.021 vs. SP600125 group (ANOVA, Bonferroni post hoc); ^*p* < 0.039 morphine group vs. SP600125 (3 mg/kg) group (ANOVA, Bonferroni post hoc); x *p* < 0.0001 vs. vehicle group (ANOVA); #*p* < 0.044 vs. SP600125 (3 mg/kg) or morphine (6 mg/kg) (ANOVA, Bonferroni post hoc)

Systemic administration of morphine (6 mg/kg), SP600125 (3 mg/kg), and their combination suppressed composite pain scores relative to the vehicle group (F_3,19_ = 215.15, *p* < 0.0001) in a time-dependent manner (F_33,209_ = 23.09, *p* < 0.0001; Fig. 2a). This suppression was observed at 5 min (acute phase 1) and from 25 to 45 min (inflammatory phase 2) post-formalin injection (*p* < 0.0001). The combination of morphine with SP600125 further reduced composite pain scores relative to treatment with SP600125 alone (*p* < 0.021 at 5 and 25–35 min post-formalin). Moreover, at 5 min post-formalin, morphine produced a greater suppression of the composite pain score than with SP600125 alone (*p* < 0.039). Analysis of the AUC of pain behavior revealed that morphine, SP600125 and their combination reduced pain behavior relative to the vehicle group for both phases (F_3,19_ = 72.33, *p* < 0.0001 (phase 1); F_3,19_ = 182.91, *p* < 0.0001 (phase 2)) of the formalin test (Fig. 2b and c). Moreover, the combination of morphine with SP600125 produced a greater antinociceptive effect than either drug given alone for the first (*p* < 0.044) and second (*p* < 0.002) phases, thereby revealing a sub-additive antinociceptive effect of the combination of morphine with SP600125 (Fig. 2b and c).

### SP600125 blocks tolerance to morphine antinociception

**Fig. 3:**
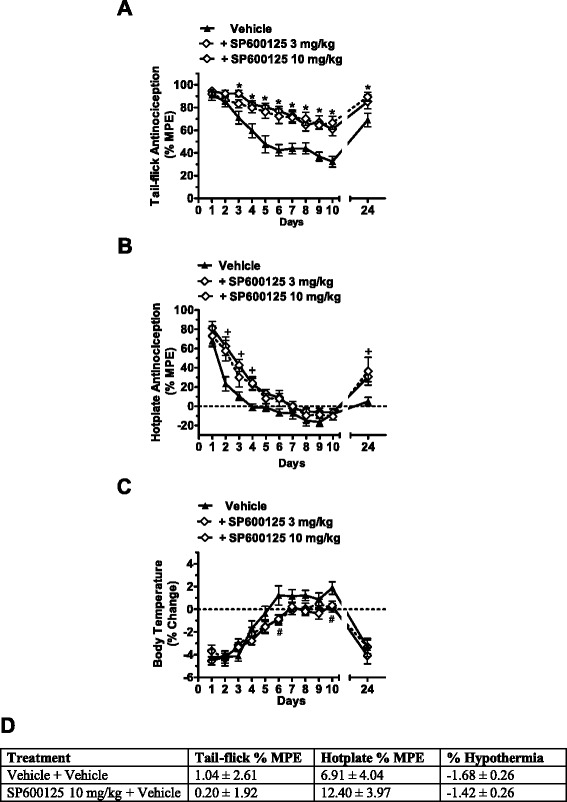
SP600125 delays tolerance for the antinociceptive effects of chronically administered morphine. Chronic tolerance to the antinociceptive effects of morphine is mediated by JNK signaling. Wild-type mice were treated with vehicle (black line with triangles), 3 mg/kg SP600125 (SP6; dashed black line with diamonds), or 10 mg/kg SP600125 (SP6; black line with diamonds) 60 min prior to administration of 10 mg/kg morphine for ten consecutive days. Treatment with either 3 mg/kg or 10 mg/kg SP600125 delayed tolerance to the effects of chronically administered morphine in the tail-flick test and the hot plate test. Treatment with either 3 mg/kg or 10 mg/kg SP600125 also reduced tolerance for the hypothermic effects of chronically administered morphine. Tail-flick antinociception (**a**), hotplate antinociception (**b**), and body temperature (**c**) were measured 60 min later. Acute treatment with SP600125 alone (3 mg/kg or 10 mg/kg) had no effect on tail-flick antinociception (*p* = 0.79), hotplate antinociception (*p* = 0.36), or hypothermia (*p* = 0.5) (**d**). Data are expressed as mean ± S.E.M. (n = 9–19 per group). *p < 0.007 for SP600125 (3 or 10 mg/kg) vs. vehicle group (ANOVA, Bonferroni post hoc); + *p* < 0.003 for SP600125 (3 or 10 mg/kg) vs. vehicle group (ANOVA, Bonferroni post hoc); #*p* < 0.016 for SP600125 (3 or 10 mg/kg) vs. vehicle group (ANOVA, Bonferroni post hoc)

Daily (i.p.) treatment with morphine alone resulted in the rapid development of tolerance to the antinociceptive and hypothermic effects of this drug (Fig. 3). Daily pretreatments with the SP600125 (3 mg/kg or 10 mg/kg) in combination with morphine prolonged tail-flick latencies relative to morphine alone (F_2, 40_ = 22.81, *p* < 0.0001; Fig. 3a) in a time-dependent manner (F_20, 400_ = 1.89, *p* < 0.012). Indeed, pre-treatment (i.p.) with SP600125 (3 mg/kg and 10 mg/kg) blunted the antinociceptive tolerance to morphine relative to the vehicle pre-treated group in the tail-flick test from day 3 (F_2, 52_ = 5.43, *p* < 0.007) to day 10 (F_2, 51_ = 12.73, *p* < 0.0001) and also on day 24 (F_2, 40_ = 4.56, *p* < 0.016). Moreover, daily pretreatment with SP600125 slowed the development of antinociceptive tolerance to morphine in the hotplate test (F_2, 24_ = 14.82, *p* < 0.0001; Fig. 3b). Both doses (3 mg/kg, *p* < 0.003 and 10 mg/kg, *p* < 0.0001) of SP600125 attenuated, but did not prevent, antinociceptive tolerance to morphine relative to vehicle-treated group in the hotplate test. Acute treatment with SP600125 alone (10 mg/kg n = 32) had no effect relative to the vehicle treated group (n = 20) on tail-flick antinociception (*p* = 0.79), hotplate antinociception (*p* = 0.36), or hypothermia (*p* = 0.5) (Fig. 3d).

### SP600125 delays tolerance to the hypothermic effects of morphine

Both 3 and 10 mg/kg (i.p.) of SP600125 (F_2, 33_ = 5.06; *p* = 0.012) delayed tolerance to the hypothermic effects of chronically administered 10 mg/kg morphine in a time dependent manner (F_20, 330_ = 5.59, *p* < 0.0001) (Fig. 3c). Pre-treatment with either dose of SP600125 (3 mg/kg and 10 mg/kg) blunted tolerance to the hypothermic effects of morphine compared to morphine-treated mice receiving vehicle pre-treatment at day 6 (F_2, 48_ = 4.14, *p* < 0.022) and day 10 (F_2, 47_ = 4.51, *p* < 0.016) (Fig. 3c).

### JNK inhibition does not affect antinociceptive tolerance to fentanyl

**Fig. 4:**
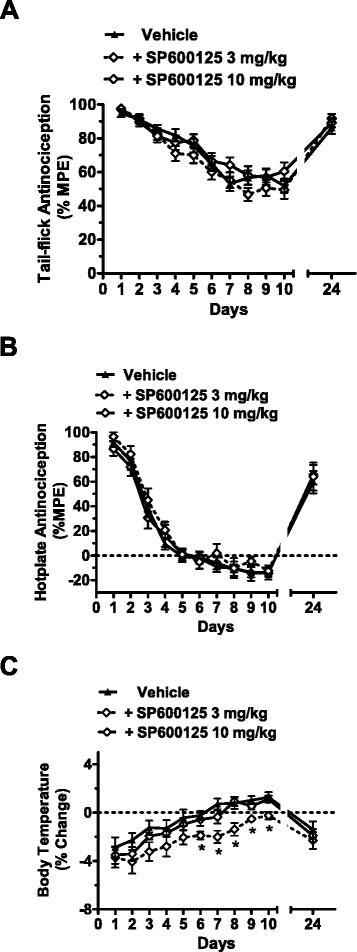
Tolerance to the antinociceptive effects of fentanyl is not blocked by SP600125. JNK inhibition attenuates chronic tolerance to the hypothermic but not the antinociceptive effects of repeated treatment with fentanyl (0.3 mg/kg). Wild-type mice were treated with vehicle (black line with triangles), 3 mg/kg SP600125 (dashed black line with diamonds), or 10 mg/kg SP600125 (black line with diamonds) 60 min prior to administration of fentanyl (0.3 mg/kg) for ten consecutive days. Tail-flick antinociception (**a**), hotplate nociception (**b**), and body temperature (**c**) were measured 60 min later. Treatment with only SP600125 (3 mg/kg) attenuated tolerance to the hypothermic effects of chronically administered 0.3 mg/kg fentanyl alone. Data are expressed as mean ± S.E.M. (n = 10–24 per group). *p < 0.0001 for SP600125 (3 mg/kg) vs. vehicle or SP600125 (10 mg/kg) groups (ANOVA, Bonferroni post hoc)

Chronic daily (i.p.) pretreatment with the JNK inhibitor SP600125 (3 mg/kg or 10 mg/kg) did not attenuate development of tolerance to the antinociceptive effects of 0.3 mg/kg fentanyl in the tail-flick test (F_2, 63_ = 2.41; *p* = 0.098; Fig. 4a) or the hotplate test (F_2, 45_ = 1.091; *p* = 0.344; Fig. 4b) at any time point (F_20, 630_ = 0.89; *p* = 0.607 for the tail-flick; F_20, 450_ = 0.41; *p* = 0.99 for the hotplate). By contrast, pre-treatment with SP600125 (F_2, 15_ = 18.93; *p* < 0.0001) did delay tolerance to the hypothermic effects of chronically administered 0.3 mg/kg fentanyl (Fig. 4c). Tolerance to the hypothermic effects of fentanyl was attenuated only by SP600125 3 mg/kg (*p* < 0.0001), but not by SP600125 10 mg/kg (*p* = 0.278) relative to fentanyl-treated rats receiving vehicle in lieu of SP600125 (Fig. 4c).

### Assessment of cisplatin-induced mechanical allodynia following administration of SP600125, morphine, and their combination

**Fig. 5:**
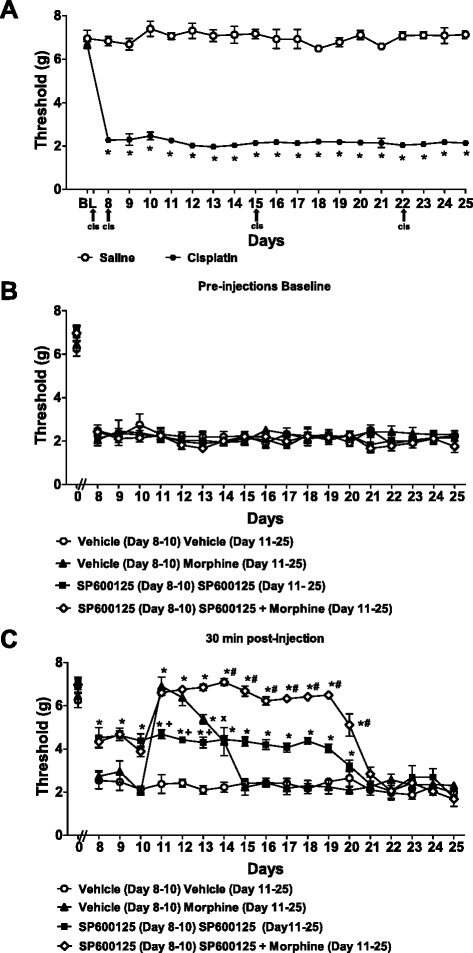
Treatment with SP600125 delays tolerance to the anti-allodynic effect of morphine on responsiveness to mechanical stimulation. Cisplatin produces time-dependent sensitization to mechanical stimulation. The time course for the development of mechanical allodynia (**a**) in cisplatin-treated relative to saline treated-mice. Baseline paw withdrawal thresholds (**b**) prior to treatment with vehicle, SP600125 (3 mg/kg i.p.), morphine (6 mg/kg i.p.) or their combination. Paw withdrawal thresholds 30 min after treatment (**c**) with these drug combinations. Data are expressed as mean ± S.E.M. (n = 5–6 per group). *p < 0.013 vs. vehicle or saline group (ANOVA, Bonferroni post hoc); + *p* < 0.006 vs. morphine or morphine with SP600125 groups (ANOVA, Bonferroni post hoc); #*p* < 0.01 vs. SP600125 given alone (ANOVA, Bonferroni post hoc); x *p* < 0.001 vs. morphine with SP600125 (ANOVA, Bonferroni post hoc)

Cisplatin decreased mechanical paw withdrawal thresholds relative to saline-treatment in mice (F_1,28_ = 2666.98, *p* < 0.0001), consistent with the development of mechanical allodynia (Fig. 5a). Mechanical allodynia was detectable in the absence of SP600125 or morphine-treatment throughout the duration of this experiment from day 8 to day 25 (*p* < 0.0001) (Fig. 5a).

Prior to SP600125 and/or morphine treatment, baseline measurements of cisplatin-induced mechanical allodynia did not differ in any group (F_3,18_ = 1.03, *P* = 0.405) at any time point (F_54,324_ = 0.81, *p* = 0.822) (Fig. 5b). Mechanical paw withdrawal thresholds assessed prior to drug injection were similar in groups of mice receiving vehicle, morphine (6 mg/kg), SP600125 (3 mg/kg) or morphine (6 mg/kg) combined with SP600125 (3 mg/kg) from day 8 (F_3,18_ = 0.93, *p* = 0.447) to day 25 (F_3,18_ = 1.88, *p* = 0.169; Fig. 5b).

Morphine (6 mg/kg; given from day 11 to day 25), SP600125 (3 mg/kg; given from day 8 to day 25), or their combination (SP600125 from day 8 to day 25 + morphine from day 11 to day 25) were chronically administered and tested for their anti-allodynic effects in cisplatin-induced neuropathic pain (Fig. 5c). Morphine (6 mg/kg), SP600125 (3 mg/kg), and their combination all suppressed cisplatin-induced mechanical allodynia relative to vehicle (F_3,18_ = 143.09, *p* < 0.0001) (Fig. 5c). Morphine (6 mg/kg), SP600125 (3 mg/kg), and their combination produced time-dependent (F_54,324_ = 14.93, *p* < 0.0001) attenuations of mechanical allodynia relative to pre-injection baselines. This attenuation of mechanical allodynia was observed relative to the vehicle group from day 8 (F_3,18_ = 8.61, *p* < 0.001) to day 20 (F_3,18_ = 14.05, *p* < 0.0001) (Fig. 5c).

Morphine treatment alone (6 mg/kg) reversed cisplatin-induced mechanical allodynia relative to vehicle from day 11 (*p* < 0.0001) to day 14 (*p* < 0.006) (Fig. 5c). Additionally, morphine (6 mg/kg) administered alone increased mechanical paw withdrawal thresholds to a greater extent than SP600125 (3 mg/kg) administered alone from day 11 (*p* < 0.009) to day 13 (*p* < 0.011). However, the anti-allodynic efficacy of morphine dissipated rapidly over time, consistent with development of tolerance to morphine. Consequently, morphine (6 mg/kg) no longer produced antinociception starting on its fifth day of administration (day 15). From day 15 to day 25, morphine (6 mg/kg) no longer produced anti-allodynic effects relative to vehicle (*p* > 0.851) (Fig. 5c). SP600125 (3 mg/kg) alone alleviated cisplatin-induced mechanical allodynia relative to vehicle from day 8 (*p* < 0.006) to day 19 (*p* < 0.0001). However, by day 19, SP600125 (3 mg/kg) no longer produced antinociception (Fig. 5c).

Morphine (6 mg/kg) combined with SP600125 (3 mg/kg) suppressed cisplatin-induced mechanical allodynia in comparison to the vehicle group from day 8 (*p* < 0.013) until day 20 (*p* < 0.001). Morphine combined with SP600125 induced paw withdrawal thresholds similar in magnitude to morphine alone on the initial day of morphine dosing (day 11; *p* = 1.000) and on day 12 (*p* = 1.000). However, the anti-allodynic effect of morphine combined with SP600125 was greater than administration of morphine or SP600125 given alone from day 13 (*p* < 0.001 for each comparison) to the day 20 (*P* < 0.01 for each comparison), when tolerance to morphine anti-allodynic efficacy was developing. From day 21 to day 25, when SP600125 treatment no longer produced antinociception, the anti-allodynic effect of morphine + SP600125 combination treatment was no longer present (F_3,18_ = 1.48, *p* = 0.447 (day 21); 0.97, *p* = 0.428 (day 22); 1.28, *p* = 0.312 (day 23); 1.55, *p* = 0.237 (day 24); 1.92, *p* = 0.163 (day 25)) (Fig. 5c). Thus, SP600125 alone produced intrinsic antinociceptive efficacy and when combined with morphine it delays tolerance to the anti-allodynic effects of morphine. This finding suggests that tolerance to effects of SP600125 on JNK signaling may serve as the basis for the eventual development of tolerance to morphine when both are co-administered.

### Assessment of cold allodynia following administration of SP600125, morphine, and their combination in cisplatin-treated mice

**Fig. 6:**
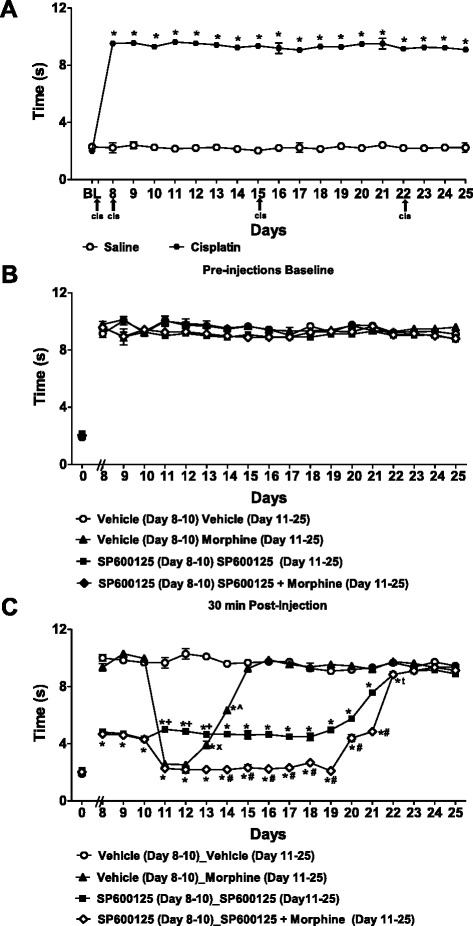
Treatment with SP600125 delays tolerance to the anti-allodynic effect of morphine on responsiveness to cold stimulation. Cisplatin produces time-dependent sensitization to cold stimulation. The time course for the development of cold allodynia (**a**) in cisplatin-treated relative to saline treated-mice. Baseline paw latencies in response to cold stimulation (**b**) prior to treatment with vehicle, SP600125 (3 mg/kg i.p.), morphine (6 mg/kg i.p.) or their combination. Paw latencies in response to cold stimulation 30 min after treatment (**c**) with these drug combinations. Data are expressed as mean ± S.E.M. (n = 5–6 per group). *p < 0.001 vs. vehicle or saline group group (ANOVA, Bonferroni post hoc); + *p* < 0.004 vs. morphine or morphine with SP600125 groups (ANOVA, Bonferroni post hoc); #*p* < 0.0001 vs. SP600125 given alone (ANOVA, Bonferroni post hoc); x *p* < 0.0001 vs. morphine with SP600125 (ANOVA, Bonferroni post hoc); ^*p* < 0.0001 vs. SP600125 alone or morphine with SP600125 groups (ANOVA, Bonferroni post hoc); t *p* < 0.001 vs. vehicle or morphine (ANOVA, Bonferroni post hoc)

Responsiveness to acetone was increased in the cisplatin-treated group relative to the saline group (F_1,28_ = 6481.82, *p* < 0.0001), consistent with development of cold allodynia (Fig. 6a). Cold allodynia was present in cisplatin-treated mice throughout the duration of this study from day 8 to day 25 (*p* < 0.0001) (Fig. 6a). Prior to drug treatment, baseline responsiveness to cold did not differ in any group (F_3,18_ = 1.64, *p* = 0.215) at any time point (F_54,324_ = 0.95, *p* = 0.569) (Fig. 6b). Response time to cold stimulation was similar in groups receiving vehicle, morphine (6 mg/kg), SP600125 (3 mg/kg, or morphine (6 mg/kg) combined with SP600125 (3 mg/kg) from day 8 (F_3,18_ = 1.75, *p* = 0.192) to day 25 (F_3,18_ = 1.45, *p* = 0.263) (Fig. 6b).

Morphine (6 mg/kg; given from day 11 to day 25), SP600125 (3 mg/kg; given from day 8 to day 25) and their combination (3 mg/kg SP600125 from day 8 to day 25 + 6 mg/kg morphine from day 11 to day 25) were chronically administered (i.p.) and tested for their anti-allodynic effect on cisplatin-induced neuropathic pain (Fig. 6c). Morphine (6 mg/kg), SP600125 (3 mg/kg), and their combination all suppressed cisplatin-induced cold allodynia relative to vehicle (F_3,18_ = 2704.06, *p* < 0.0001) (Fig. 6c). Morphine (6 mg/kg), SP600125 (3 mg/kg), and their combination also produced time-dependent (F_54,324_ = 201.45, *p* < 0.0001) attenuation of cold allodynia relative to pre-injection baselines. The attenuation of cold allodynia induced by SP600125 alone or the combination of morphine and SP600125 was observed relative to the vehicle group from day 8 (F_3,18_ = 212.64, *p* < 0.0001) to day 22 (F_3,18_ = 15.87, *p* < 0.0001) (Fig. 6c).

Morphine treatment alone (6 mg/kg) reversed cisplatin-induced cold allodynia relative to vehicle from day 11 (*p* < 0.0001) to day 14 (*p* < 0.0001) (Fig. 5c). Additionally, morphine administered alone lowered time responses to cold stimulation to a greater extent than SP600125 administered alone from day 11 (*p* < 0.0001) to day 13 (*p* < 0.004). However, morphine’s anti-allodynic effect dissipated over time, consistent with development of tolerance to morphine. Consequently, morphine no longer produced antinociception starting on its fifth consecutive day of administration (day 15). Indeed, from day 15 to day 25, morphine (6 mg/kg) no longer produced anti-allodynic effect relative to vehicle (*p* > 0.164) (Fig. 6c).

Interestingly, SP600125 (3 mg/kg) alone alleviated cisplatin-induced cold allodynia relative to vehicle from day 8 (*p* < 0.0001) to day 22 (*p* < 0.001). On day 14, SP600125 given alone showed lower (*p* < 0.0001) time responses to cold stimulation than morphine given alone (Fig. 6c).

Morphine (6 mg/kg) combined with SP600125 (3 mg/kg) suppressed cisplatin-induced cold allodynia in comparison to the vehicle group from day 8 (*p* < 0.0001) to day 22 (*p* < 0.001). Morphine combined with SP600125 lowered time responses to cold stimulation with a similar magnitude as morphine alone on day 11 (*p* = 1.000) and on day 12 (*p* = 1.000). The anti-allodynic effect of morphine combined with SP600125 was greater than the anti-allodynia seen with administration of morphine or SP600125 given alone from day 13 (*p* < 0.0001 for each comparison) to day 21 (*p* < 0.0001 for each comparison). On day 22, mice receiving the combination of morphine with SP600125 showed a similar response to cold stimulation (*p* = 1.000) as did mice receiving SP600125 alone, and both groups had significantly lowered responses to cold stimulation relative to vehicle/morphine groups (*p* < 0.001). Thus, anti-allodynic efficacy of the combination treatment of SP600125 with morphine was apparent during the interval when SP600125 produced antinociception and tolerance to morphine anti-allodynic efficacy was developing. From day 23 to day 25, the anti-allodynic effect was no longer observed in any treatment group (F_3,18_ = 1.10, *p* = 0.376 (day 23); 0.62, *p* = 0.610 (day 24); 2.32, *p* = 0.110 (day 25)), Fig. 6c).

## Discussion

In this study, morphine antinociception was measured in multiple assays using the formalin, tail-flick, and hotplate tests, and also by measuring suppression of mechanical and cold allodynia in a chemotherapy-induced neuropathic pain model. We found that SP600125 produces dose-dependent antinociceptive effects in the formalin test. Our results in the formalin test demonstrate that co-administration of SP600125 (3 and 10 mg/kg) and morphine (6 mg/kg) produces a sub-additive antinociceptive effect compared to treatment with either morphine (6 mg/kg) or SP600125 (3 mg/kg) alone. We also found that pre-treatment with SP600125 (3 and 10 mg/kg) slows the development of tolerance to the antinociceptive effects of morphine. During a ten day treatment course, SP600125 pre-treatment prevented the development of complete tolerance to morphine-induced tail-flick antinociception (five day delay before any evidence of tolerance was detected) and a one day delay in tolerance to morphine induced hotplate antinociception. Consistent with previous work, we found that tolerance for the antinociceptive effect of fentanyl was not affected by SP600125, providing additional evidence of functionally selective mechanisms of opioid tolerance. Finally, SP600125 (3 mg/kg) alone attenuated mechanical and cold allodynia in cisplatin-treated mice. Strikingly, SP600125 (3 mg/kg) pre-treatment prolonged the anti-allodynic effect of morphine by several days (5 and 7 days for mechanical and cold, respectively) depending on the modality. These results demonstrate that JNK signaling plays a crucial role in directly mediating antinociception but is also involved in chronic tolerance to morphine antinociception in acute, inflammatory and neuropathic pain assays.

A noteworthy observation from our studies is that inhibition of JNK through systemic SP600125 (i.p.) administration produces antinociception in an inflammatory pain model (formalin test) in a dose-dependent manner. Indeed, our study demonstrates that 3 mg/kg of SP600125 produced the greatest antinociceptive effect compared to other doses (0.1, 1 and 10 mg/kg i.p.) in the formalin test. Likewise, it also seems that 3 mg/kg SP600125 is the better dose compared to 10 mg/kg for preventing tolerance to morphine in the hotplate and tail-flick tests. However, further studies outside the scope of the current manuscript would be necessary to confirm conclusively whether the shape of the dose–response curve for the effect of SP600125 on morphine tolerance and antinociception are bell or sigmoidally shaped. The alleviation of pain by SP600125 in our inflammatory pain model corroborates a previous study reporting that local peripheral administration of SP600125 produces antinociceptive effects in melittin (principal toxic peptide from whole bee venom)-induced nociception [23]. One study found that formalin increased expression of phosphorylated MAPKs (mitogen-activated protein kinases) in the spinal cord including phospho-p38, phospho-ERK and phospho-JNK [24]. While yet another study demonstrates the beneficial effect of SP600125 on pain (weight bearing) in an arthritis model [25]. The attenuation of the nociceptive response of SP600125 following intrathecal administration in the formalin test has been investigated [26]. However, our study is the first to demonstrate that systemic administration of JNK inhibitor (SP600125 at 3 mg/kg) combined with morphine (6 mg/kg) produces a sub-additive antinociceptive effect in the formalin test. One possible explanation for this sub-additive antinociceptive effect when morphine and SP600125 are combined is that SP600125 drives enhanced antinociception by decreasing the activation of peripheral MAPKS such as phospho-JNK [24].

Our study also demonstrates that two doses (3 or 10 mg/kg) of JNK inhibitor (SP600125) attenuated chronic tolerance to the antinociceptive effects of morphine in the tail-flick and hotplate tests. This finding corroborates previous studies demonstrating that JNK signaling mediates chronic tolerance for the antinociceptive effects of morphine in the tail-flick test [15, 21]. Interestingly, SP600125 (3 mg/kg) pre-treatment prolonged the maximal antinociceptive effect of morphine by several days (3 and 8 days for hotplate and tail-flick tests, respectively) depending on the assay. Previous work using the hotplate assay revealed a learning phenomenon that causes basal response latencies to decrease during repeated testing [27]. Since our experiment involved repeated daily testing of mice using the hotplate, it is possible that the diminishment in hotplate response observed could be due to the mouse learning to exhibit certain behaviors in order to be removed from the hotplate apparatus [28, 29]. Thus, we would likely observe a more pronounced effect of SP600125 on hotplate antinociception if daily experiments were obtained from separate cohorts of hotplate-naïve, morphine-treated mice.

**Fig. 7:**
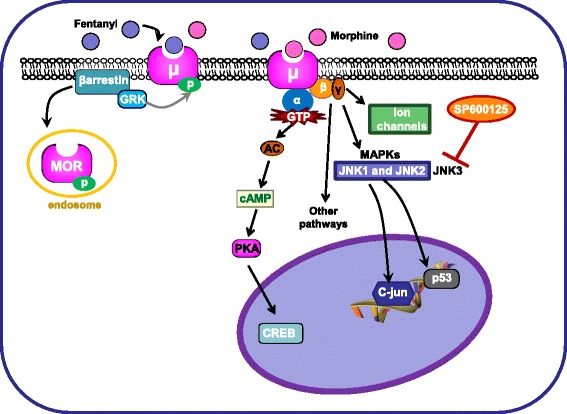
Schematic representation of different hypotheses/mechanisms involved in the development of tolerance to the antinociceptive effect of fentanyl and morphine. Down-regulation and desensitization of MOR represent two possible biochemical processes that could underlie JNK-mediated morphine tolerance *in vivo*. The findings of our study and others suggests that tolerance for the antinociceptive effects of morphine is mediated by JNK signaling, possibly through desensitization of MOR, defined for this study as the functional uncoupling of the receptor from its G protein signaling components. In contrast, inhibition of JNK does not alter tolerance for the antinociceptive effects of fentanyl. Tolerance for the antinociceptive effects of fentanyl appears to be mediated by a classic mechanism involving GRK phosphorylation of MOR followed by βarrestin2 recruitment

In acute pain tests, chronic tolerance to morphine, a modestly efficacious and weakly internalizing MOR agonist, is mediated primarily by JNK while tolerance to the more efficacious and strongly internalizing opioid, fentanyl is not [4, 15, 21] (Fig. 7). Thus, antinociceptive tolerance for strongly internalizing (e.g., fentanyl) MOR ligands may be mediated by a ‘classical’ GRK/βarrestin mechanism while tolerance for weakly internalizing ligands (e.g., morphine) is likely to be mediated by JNK signaling mechanisms, potentially in a cell-type specific manner [4, 15, 21] (Fig. 7).

One surprising result from this study was the finding that JNK inhibition attenuated hypothermic tolerance to fentanyl. Based on previous work examining the role of JNK in acute tolerance for the analgesic effects of fentanyl, we did not expect SP600125 treatment to affect tolerance for any of the physiological effects of fentanyl [20]. Our results that SP600125 does appear to affect tolerance for the hypothermic effect of morphine and fentanyl raises the possibility that divergent cell-type specific tolerance mechanisms might exist for the antinociceptive compared to thermoregulatory effects of fentanyl. For example, JNK-mediated tolerance might occur in thermoregulatory neurons of the preoptic anterior hypothalamus (POAH) that express MOR and mediate the hypothermic effects of morphine and fentanyl [30].

This study is the first to demonstrate that systemic administration of JNK inhibitor (SP600125 at 3 mg/kg) suppresses mechanical and cold allodynia in a chemotherapy-induced neuropathic pain model using cisplatin. Our finding corroborates previous research that demonstrated intrathecal administration of SP600125 produces anti-allodynic effects in a spinal nerve ligation model of neuropathy [31]. Interestingly, the anti-allodynic effects of morphine are greater than those of SP600125 administered alone and diminishment of the morphine-specific anti-allodynic component is delayed by pre-treatment with SP600125. However, we found that treatment with SP600125 alone does not have significant effects on either tail-flick or hotplate antinociception, possibly due to signaling changes induced by toxic neuropathy or differences in the neuronal cell-types and/or signaling pathways associated with the different pain states or testing paradigms. JNK inhibition alone appears to be ineffective at alleviating acute (tail-flick and hotplate) pain, although we did observe antinociception in inflammatory and chemotherapy-induced neuropathic pain models. It has been demonstrated previously that many antinociceptive compounds are more effective in chronic pain [32] relative to acute pain models.

We also find that pre-treatment with SP600125 significantly delays tolerance to the anti-allodynic effects of morphine for both mechanical and cold allodynia in chemotherapy-induced peripheral neuropathy in cisplatin-treated mice. Our work supports previous studies showing that JNK signaling is essential for tolerance to morphine in sciatic nerve injury-induced neuropathic pain [22]. However, we extend the role of JNK-mediated morphine tolerance to a highly clinically relevant model of chemotherapy-induced neuropathy by demonstrating that JNK mediates morphine tolerance in a chemotherapy-induced pain model.

The precise mechanisms through which JNK mediates tolerance to morphine are still being elucidated. However, down-regulation and/or desensitization of MOR represent two possible biochemical processes that could underlie JNK-mediated tolerance *in vivo* (Fig. 7). Previous work has shown that treatment with SP600125 prevents the reduction of D-Ala^2^-Met^5^-Glyol-enkephalin (DAMGO)-stimulated [^35^S] sGTPγS binding in spinal cord homogenates that is caused by repeated treatment with morphine, but doesn’t affect down regulation caused by repeated treatment with fentanyl [20]. Subsequent work has shown that JNK facilitates desensitization of voltage-gated calcium channel inhibition in DRG neurons [33]. These findings indicate that desensitization of MOR, defined for this study as the functional uncoupling of the receptor from its G protein signaling components, is at least partially responsible for JNK-mediated tolerance for the antinociceptive effects of morphine (Fig. 7). Despite this evidence, it is important to note that tolerance to MOR agonists can be influenced by a number of other pharmacodynamic processes including, but not limited to, NMDA receptor modulation [15], nitric oxide signaling pathways (which can include JNK) [16], and protein kinase C activity [17].

## Conclusions

The present study provides direct evidence that chronic tolerance to morphine is mediated at least partially by a JNK mechanism in acute, inflammatory and neuropathic pain states. Indeed, we found that inhibition of JNK, using SP600125, produced a dose-dependent antinociceptive effect in the formalin test. Moreover, combination treatment with SP600125 and morphine produced a sub-additive antinociceptive effect. In the assessment of JNK inhibition on acute pain, we observed that SP600125 attenuates chronic tolerance to the antinociceptive effects of morphine, but not fentanyl in the tail-flick and hotplate tests. This supports previous reports that chronic tolerance for opioids occurs in an agonist specific manner suggesting that functional selectivity extends to GPCR desensitization mechanisms and tolerance pathways. SP600125 also attenuated cisplatin-induced mechanical and cold allodynia in a chemotherapy-induced toxic neuropathy model. Strikingly, SP600125 pre-treatment prolonged the anti-allodynic effect of morphine by several days (5 and 7 days for mechanical and cold, respectively). These results demonstrate that JNK signaling plays a crucial role in mediating antinociception and chronic tolerance to morphine in acute, inflammatory and neuropathic pain states.

## Methods

### Subjects

Experiments were performed using wild-type C57Bl6/J mice obtained from Jackson Laboratories (Bar Harbor, Maine). Mice used in these experiments were housed under a 12:12 h light–dark cycle (lights on 07:00, lights off 19:00) and provided with standard mouse chow *ad libitum*. All animal care and experimental procedures used in this study were approved by the Institutional Animal Care and Use Committee of the Penn State University College of Medicine or Indiana University Bloomington and conform to the Guidelines of the National Institutes of Health on the Care and Use of Animals.

### Drugs

Morphine sulfate was purchased from Sigma Aldrich (St. Louis, MO) and was also obtained from National Institute on Drug Abuse Drug Supply (Bethesda, MD). Fentanyl was obtained from the National Institute on Drug Abuse Drug Supply (Bethesda, MD). SP600125 was obtained from Sigma-Aldrich (St. Louis, MO) [19]. Cisplatin and 4 % sodium bicarbonate (NaHCO_3_ dissolved in 0.9 % NaCl) were purchased from Tocris (Ellisville, MO). Morphine, fentanyl, cisplatin and sodium bicarbonate were dissolved in normal saline (0.9 % NaCl in water) for *in vivo* administration. A 25 mg/ml stock solution of SP600125 was prepared in 100 % DMSO and was subsequently diluted in vehicle containing 0.9 % saline, 5 % Kolliphor EL, 5 % ethanol (18:1:1 vehicle) for *in vivo* administration. The total amount of DMSO contained in all SP600125 and corresponding vehicle injections was 4 % (v/v). Doses of morphine and SP600125 were chosen that were previously shown to be efficacious in tail-flick, hotplate, and chemotherapy-induced neuropathic pain models [19–22, 34]. SP600125 was stored at −20 °C whereas morphine, fentanyl, cisplatin and sodium bicarbonate were stored at room temperature. The drugs or vehicle were prepared fresh on the day of the experiment and administered intraperitoneally (i.p.; SP600125) or subcutaneously (s.c.; morphine or fentanyl) in a single volume of 10 ml/kg of body weight.

### Procedures

First, the antinociceptive effect of different doses of SP600125 (0.1, 1, 3 and 10 mg/kg i.p.) were assessed relative to vehicle using the formalin test (2.5 %). Second, the antinociceptive effect of SP600125 in combination with morphine was determined in another group of mice to evaluate the presence of a greater antinociceptive effect when these drugs were given together.

Third, tail-flick and hotplate antinociception as well as hypothermia were measured in different mice receiving either sub-cutaneous (s.c.) injections of morphine (10 mg/kg × 10 days) or fentanyl (0.3 mg/kg × 10 days) [20]. One hour prior to administration of morphine or fentanyl, mice were pretreated with intraperitoneal (i.p.) injections of either vehicle, 3 mg/kg SP600125, or 10 mg/kg SP600125. Daily measurements of body temperature and tail-flick and hotplate responses were recorded before administration of any vehicle or drug (pre-test) and one hour after treatment with morphine or fentanyl. Tolerance to morphine or fentanyl was examined in mice treated chronically for ten consecutive days with morphine or fentanyl alone or in combination with SP600125. Two weeks after cessation of drug treatment, recovery from tolerance to the antinociceptive effect of morphine was assessed in the tail-flick and hotplate tests following pre-treatment with vehicle, SP600125 (3 mg/kg), or SP600125 (10 mg/kg) given 60 min prior to a challenge dose of morphine or fentanyl.

Fourth, a separate cohort of mice was used to measure mechanical and cold allodynia in cisplatin-treated mice receiving either vehicle only (day 8–25), morphine (6 mg/kg, day 11–25) only, SP600125 (3 mg/kg, day 8–25) only, or SP600125 (3 mg/kg, day 8–25) pre-treatment 30 min prior to subsequent treatment with vehicle or morphine (6 mg/kg, day 11–25).

### Formalin test

The formalin test is a well-established model of persistent pain characterized by a transient, biphasic pattern of pain behaviour. The early phase is characterized by acute activation of C and Aδ fibers. The late phase involves an inflammatory reaction in peripheral tissue [35], the development of central nervous system sensitization [36, 37], and additionally involves activation of primary afferent nociceptors [38]. Mice were acclimatized to the testing environment (clear Plexiglass box 10 x 10 x 10 cm) during 15 min or until cessation of exploratory behaviour. Mice were injected intraperitoneally (i.p.) with either SP600125 (0.1, 1, 3, 10 mg/kg), morphine (6 mg/kg) or their combination (SP600125 3 mg/kg + morphine 6 mg/kg). Following each injection, the mice were immediately placed back in the observation chamber. Nociceptive behaviour was observed with the help of a mirror angled at 45° below the observation chamber. Observation of the animal’s behaviour was performed in consecutive 5-min periods for 60 min following administration of 2.5 % formalin (10 μl). In each 5-min bin, the total time the animal spent in three different behavioural categories was recorded: (0) the injected paw has little or no weight placed on it; (1) the injected paw is raised; (2) the injected paw is licked, shaken or bitten. Nociceptive behaviour was quantified using the composite pain score-weighted scores technique (CPS-WST_0,1,2_) [39], where each pain behaviour is weighted by the amount of time spent in each category (0,1,2). The area under the curve (AUC), which corresponds to CPS-WST_0,1,2_ x time (min) was calculated for the acute phase (0–15 min; Phase 1) and the inflammatory phase (15–60 min; Phase 2) using the trapezoidal rule.

### Assessment of tail-flick and hotplate antinociception

Tail-flick antinociception was measured using a Columbus Instruments TF-1 tail-flick analgesia meter (Columbus, OH). The radiant heat source on the apparatus was calibrated to elicit a tail-flick latency of 3–4 s in untreated wild-type control mice. A 10 s cutoff was used for all tail-flick tests to avoid tissue damage to the tail. Tail-flick response latencies were measured in a single cohort of animals daily for ten consecutive days and also on day 24, before administration of SP600125 and morphine or fentanyl (pre-test) and 60 min after treatment with fentanyl or morphine (post-test).

Hotplate antinociception testing was measured using a hotplate testing meter from Columbus Instruments set to 55 °C (Columbus, OH). A 30 s cutoff was used to avoid paw tissue damage. The hotplate response latencies were measured in a single cohort of animals for ten consecutive days and also on day 24, both before administration of any drugs and also 60 min after treatment with morphine or fentanyl. The hotplate and tail-flick analgesic responses were calculated as the percentage the maximal possible effect (%MPE) with %MPE = (post-drug latency – pre-drug latency)/(10 – pre-drug latency) × 100 for tail flick analgesia and %MPE = (post-drug latency – pre-drug latency)/(30- pre-drug latency) for hotplate antinociception.

### Measurement of body temperature

Body temperature was measured using a mouse rectal thermometer (Physitemp, Clifton, NJ) to assess possible drug-induced hypothermia. Body temperature was measured in a single cohort of animals daily for ten consecutive days and also on day 24 before drug administration and also at 60, 120, 180, and 240 min after morphine or fentanyl injection.

### Development of neuropathy

Cisplatin was administered intraperitoneally (i.p.) once a week at a dose of 5 mg/kg for 25 days (cumulative dose: 20 mg/kg i.p.) [40]. Cisplatin was diluted in normal saline (0.9 % NaCl) and delivered in a volume of 10 ml/kg body weight. Control groups were injected with an equivalent volume of saline (i.p. injection) in lieu of cisplatin [34, 41]. Before each cisplatin/saline injection, a 4 % solution of sodium bicarbonate (NaHCO_3_ dissolved in 0.9 % NaCl) [41] was administered subcutaneously (1 ml). Injections were always performed after completion of mechanical and cold withdrawal testing.

### Assessment of mechanical allodynia

Mechanical withdrawal thresholds were assessed using a digital Electrovonfrey Anesthesiometer (IITC Life Sciences, Woodland Hills, CA) equipped with a semi-flexible tip as described previously [34, 41, 42]. Mice were placed in individual plastic cages on an elevated wire mesh platform, and were allowed to habituate to the testing apparatus for at least 30 min until exploratory behavior was no longer observed. Force was applied to the midplantar region of each hind paw in each study by the same experimenter. Stable baseline responses were obtained prior to experimental testing. Mechanical stimulation was terminated upon paw withdrawal; consequently, there was no upper threshold limit set for termination of a testing trial. Paw withdrawal thresholds were assessed in duplicate for each paw. Mechanical withdrawal thresholds were measured over 25 days. Testing took place on day 0 and daily from day 8 to 25 for all animals.

### Assessment of cold allodynia

Cold allodynia was measured by applying a drop of acetone to the plantar surface of the hind paw as previously described [34, 42]. Mice were placed in individual plastic cages on an elevated platform and were habituated for at least 30 min until exploratory behaviors ceased. Acetone was loaded into a 1 mL syringe with no needle. Air bubbles were cleared from the syringe prior to acetone application. One drop of acetone (approximately 20 μl) was applied through the mesh platform onto the plantar surface of the hind paw. Care was taken to gently apply the bubble of acetone to the skin on the paw without inducing mechanical stimulation through contact of the syringe barrel with the paw. Time spent attending to the acetone-stimulated paw was measured over a 60 s observation period after acetone application was recorded. Paw withdrawal was sometimes associated with a secondary response with the animal, such as rapid flicking of the paw, chattering, biting, and/or licking of the paw. Testing order alternated between paws (i.e., right and left) until five measurements were taken for each paw. An interstimulation interval of approximately 5 min was allowed between testing of right and left paws. Cold allodynia testing took place on day 0 and daily from day 8 to 25 for all animals.

### Data analysis and statistics

All experiments were conducted in a blinded manner. Animals were randomly assigned to experimental conditions. Pain behaviour for each treatment group was expressed as mean ± SEM. Paw withdrawal thresholds (mechanical) and latencies (cold) were calculated for each paw and averaged. Data were analyzed using analysis of variance (ANOVA) for repeated measures or one-way ANOVA as appropriate. The Greenhouse-Geisser correction was applied to all repeated factors; degrees of freedom reported for significant interactions are the uncorrected values. The source of significant interactions was further evaluated by performing one-way ANOVAs at each individual time point, followed by Bonferroni post hoc tests. The different components of the total variation were settled *a priori* using multiple regression analysis [43]. Analyses were performed using SPSS statistical software (version 21.0; SPSS Incorporated, Chicago, IL, USA). *P* < 0.05 was considered significant.

## Abbreviations

AC: Adenylyl cyclase
ANOVA: Analysis of variance
AUC: Area under the curve
BL: Baseline
β-arrestin: β-arrestin 2
cAMP: Cyclic adenosine monophosphate
Ca^2+^: Calcium
cis: Cisplatin
min: Minutes
CREB: Cyclic amp-response element binding protein
C-jun: C-jun transcription factor
GRK: G Protein-coupled Receptor Kinase
JNK: c-Jun N-terminal kinase
α, β and γ: g-proteins
GTP: Guanosise triphosphate
MAPK: Mitogen-activated protein kinase
MOR: μ-opioid receptor
NaCl: Sodium chloride
NaHCO3: Sodium bicarbonate
p: Phosphorylated
p53: p53 protein
PKA: Protein kinase A
s: Seconds
SP600125: JNK inhibitor for JNK1, 2 and 3
